# Prevention of Intramammary Infections by Prepartum External Application of a Teat Dip Containing Lactic Acid Bacteria with Antimicrobial Properties in Dairy Heifers

**DOI:** 10.3390/pathogens9040288

**Published:** 2020-04-16

**Authors:** Jan-Hendrik Paduch, Johanna Lücking, Elisabeth Mansion-de Vries, Claudia Zinke, Nicole Wente, Volker Krömker

**Affiliations:** 1 Department of Bioprocess Engineering and Microbiology, University of Applied Sciences and Arts Hannover, D-30453 Hannover, Germany; paduch@ba-plauen.de (J.-H.P.);; 2 Section for Production, Nutrition and Health, Department of Veterinary and Animal Sciences, Faculty of Health and Medical Sciences, University of Copenhagen, DK-1870 Frederiksberg C, Denmark

**Keywords:** lactic acid bacteria, dairy heifers, intramammary infections, prevention

## Abstract

The aim of the current study was to investigate the effects of the prepartum external treatment of teats with a combination of four lactic acid bacteria strains *viz. Lactobacillus* (*Lb.*) *rhamnosus* ATCC 7469, *Lactococcus lactis* subsp. *lactis* ATCC 11454, *Lb. paracasei* 78/37 (DSM 26911), and *Lb. plantarum* 118/37 (DSM 26912) on the postcalving udder health of dairy heifers. The study used a split-udder design. Two weeks before the expected calving date, one of two contralateral teats of a teat pair was dipped with an aqueous suspension of lactic acid bacteria (final bacterial counts 8.40–8.47 log_10_-transformed CFU/mL) once in a week until calving; the other teat of the pair was not treated. After calving, quarter foremilk samples were taken and investigated cyto-microbiologically. In total, 629 teat pairs of 319 heifers were included. There was an association between the treatment and intramammary infections caused by the major udder-pathogenic bacteria *Staphylococcus aureus*, *Streptococcus dysgalactiae,* and enterococci, as well as clinical mastitis in the first 100 days after calving. The present study indicates that intramammary infections with major pathogens and clinical mastitis may be prevented by regular prepartum external application of lactic acid bacteria in dairy heifers.

## 1. Introduction

‘Bovine mastitis’ is one of the most important diseases in modern high-yielding dairy herds [1], causing annual economic losses of up to approximately € 1.4 billion in Germany [2]. Heifer mastitis can cause a reduction of the life milk yield of dairy cows and affect culling risk [3,4].

Most of the mastitis-causing microorganisms invade the mammary parenchyma through the teat canal [5]. Krömker and Friedrich found that in heifers, 77% of intramammary infections at parturition established in the previous period [3]. The authors concluded that the opening of the teat canal before calving is associated with heifer mastitis. In addition, the incidence of clinical mastitis during the first lactation is influenced largely by the duration of infection ante partum and the mastitis pathogen involved [3]. In New Zealand, Parker et al. observed a precalving gland prevalence of intramammary infections (IMIs) of 15.5% in dairy heifers [6]. De Vliegher et al. in their article stated that the prevalence of precalving IMI ranges from 29% to 75% of quarters in heifers and the prevalence of postcalving from 12% to 57% or higher [4]. Piepers et al. found that at least one quarter of approximately 80% of fresh heifers was culture-positive [7]. They identified nonaureus staphylococci (NAS) as the most prevalent pathogens, followed by aesculin-positive streptococci and *Staphylococcus* (*Staph.*) *aureus*. However, the study of De Vliegher et al. indicates that the presence of NAS have a protective effect against IMI with major pathogens after calving [8].

As a major risk for heifer mastitis, the premature loss of the keratin plug of the teat canal is suggested [9]. The precalving infusion of a bismuth subnitrate-based teat sealant reduces the prevalence of postcalving IMI and the incidence of clinical mastitis [10]. Sampimon et al. showed that the prepartum application of dry cow antibiotics could be a suitable approach to reduce the prevalence of mastitis-causing pathogens at calving and the following 10 to 14 days [11]. Lopez-Benavides et al. found that the precalving application of an iodine-based teat sanitizer results in the reduction of the prevalence of IMI caused by *Streptococcus* (*Strep.*) *uberis* in the first week of calving [12].

Recently, the applications of probiotic bacteria in livestock farming have been discussed as a novel approach to prevent bovine mastitis and to reduce the use of antibiotics [13,14]. Some selected strains of lactic acid bacteria are capable of inhibiting the growth of mastitis-causing pathogens in vitro [15,16,17]. Klostermann et al. found that the efficiency of *Lactococcus* (*Lc.*) *lactis* DPC3147 may be equivalent to the antibiotic therapy in the treatment of mastitis [18]. Furthermore, several other studies showed that the infusion of lactic acid bacteria into the bovine udder may stimulate the host immune response [19,20]. The aim of the present study was to investigate the effects of the prepartum application of a teat dip containing a combination of the four lactic acid bacterial strains viz. *Lactobacillus* (*Lb.*) *rhamnosus* ATCC 7469, *Lb. paracasei* 78/37 (DSM 26911), *Lb. plantarum* 118/37 (DSM 26912), and *Lc. lactis* subsp. *lactis* ATCC 11454 on the udder health of dairy heifers after calving. The antimicrobial properties of the used strains were described by Diepers et al. [17].

## 2. Results

In total, 629 teat pairs from 319 dairy heifers were included in the study. Of the treated quarters, 261 teats were treated once, 255 teats twice, and 113 teats minimum thrice (and up to maximum five times).

After calving, no mastitis-causing pathogen could be isolated from 585 untreated (93.0%) and 595 treated quarters (94.6%), respectively (Table 1). In each treatment group (untreated, treated), NAS were isolated from each 22 quarters (each 3.5%) as the most prevalent mastitis-causing pathogens, and *Staph. aureus* was detected in 19 untreated (3.0%) and 11 treated quarters (1.8%), respectively.

The somatic cell counts were recorded in the range between 3.48 and 7.29 log_10_ (somatic cells/mL) (median: 5.20 log_10_ (somatic cells/mL)) at point C1, and in the range between 3.30 and 7.33 log_10_ (somatic cells/mL) (median: 4.72 log_10_ (somatic cells/mL)) at point C2. At C1, the somatic cell count of quarter foremilk samples from 378 untreated and 363 treated quarter was ≥100,000 somatic cells/mL, and at C2, 213 untreated and 221 treated quarters showed a somatic cell count of ≥100,000 somatic cells/mL.

In the first 100 days of calving, 22 cases of clinical mastitis were reported in 22 untreated teats of 19 heifers, 10 cases in teats treated once in 10 teat pairs of 9 heifers, and one case was reported in the group of teats treated twice. In one heifer, clinical mastitis occurred in both quarters of a teat pair, of which one teat was treated only once.

The IMIs caused by the major pathogens *Staph. aureus*, *Strep. dysgalactiae,* and enterococci as well as clinical mastitis in the first 100 days of lactation were associated with the frequency of treatments. The odds ratio for IMIs caused by major pathogens was 14.5 (95% CI: 1.8–114.7) in quarters treated mostly once compared to those treated at least twice, and the odds ratio for clinical mastitis was 13.6 (95% CI: 1.8–102.9) in quarters treated mostly once. At C1, the detection of major pathogens was associated with the frequency of treatments (no or 1 × precalving application vs. ≥ 2 × precalving applications, *p* = 0.003); at C2, the association tended to be statistically significant (*p* = 0.076). There were not found any association between the application of the teat dip and both IMIs and log_10_-transformed somatic cell counts (*p* > 0.05). In addition, the farm was not associated with the outcomes.

## 3. Discussion

The aim of the present study was to investigate the effects of the prepartum external application of a teat dip containing live lactic acid bacteria in dairy heifers. The results show that the treatment of udder quarters prevents IMIs with the major pathogens *Staph. aureus*, *Strep. dysgalactiae,* and *Enterococcus* spp. in the first two weeks of the lactation as well as clinical mastitis in the first 100 days of lactation.

Due to the relevance of heifer mastitis in dairy farms [4], the study emphasized the investigation of the effect of the prepartum application of a teat dip on the postcalving udder health in dairy heifers. The split-udder design of this study allowed eliminating an individual animal effect [21]. According to Diepers et al., the used combination of all the four lactic acid bacteria *viz. Lb. rhamnosus* ATCC 7469, *Lc. lactis* subsp. *lactis* ATCC 11454, *Lb. paracasei* 78/37 (DSM 26911), and *Lb. plantarum* 118/37 (DSM 26912) was capable of inhibiting the growth of the (facultatively) pathogenic bacteria such as *Staph. aureus*, *Staph. epidermidis*, *Staph. xylosus*, *Strep. uberis*, *Strep. Agalactiae,* and *E. coli* under in vitro conditions [17]. Furthermore, these lactic acid bacterial strains were capable of adhering to bovine teat canal epithelial cells. The authors proposed that lactic acid bacteria have the potential to be explored as probiotics in preventing and treating IMIs.

The NAS were the most frequently isolated pathogens, followed by the major pathogens *Staph. aureus*, *Strep. dysgalactiae,* and enterococci. The major pathogens were isolated from a small group of quarters (2.7% of the subject quarters). Piepers et al. found that in approximately 80% of heifers, pathogens could be isolated from at least one quarter [7]. The NAS and aesculin-positive streptococci were identified as most frequent pathogens. In Germany, Krömker and Friedrich isolated NAS (13% of quarters) and *Staph. aureus* (2%) as the most frequent microorganisms from foremilk samples in the first five days of calving [3]. They reported that, in heifers, most of the IMIs at calving are established in the early period. In addition, teat skin and teat canal of heifers may act as a reservoir, especially, for staphylococci [8,22,23,24,25]. Despite the high proportion of udder quarters with somatic cell counts ≥ 100,000 somatic cells/mL (at C1 58.9% of all quarters, at C2 34.5%), intramammary infections were detected in only 6.2% (78/1258; Table 1) of all quarters. This could be due to the conservative definition of intramammary infections (detection of the same pathogen at day 7 ± 2 and day 14 ± 2 after calving) according to Kiesner et al. [26]. Fifty nine percent of streptococcal IMI and 69% of coliform IMI had a duration of at maximum 30 days during lactation [27].

In general, lactic acid bacteria produce certain metabolites such as organic acids, hydrogen peroxide, diacetyl, bacteriocins, and other substances, which may inhibit the growth of other microorganisms [28,29]. Bouchard et al. concluded that strains with high adhesion and invasion potential may compete with pathogens to colonize the bovine mammary gland tissue [16]. In the current study, the strains of lactic acid bacteria were applied topically on the teat, and therefore it can be hypothesized that the lactic acid bacteria may be established on teat skin and teat canal epithelium. Subsequently, the lactic acid bacteria may compete with mastitis-causing pathogens, which contaminate or are able to colonize teat epithelia [12,22,24,30,31], and as a consequence, may reduce the pathogen burden on the mammary gland. Interestingly, in the present study, the application of lactic acid bacteria did not affect the IMIs caused by minor-pathogenic NAS, which indicates that on teat epithelia, NAS that are a part of “normal” skin flora [32,33] could outcompete the selected strains of lactic acid bacteria. However, our findings show that a regular application may be essential to establish the lactic acid bacteria on teat epithelia and to achieve a medium- to the long-term protective effect on udder health. Since the milk samples from mastitic quarters were not investigated to identify mastitis-causing pathogens, the association between IMIs at the beginning of lactation and the clinical cases of mastitis during lactation could not be determined. Krömker and Friedrich found that in heifers, about 85% cases of clinical mastitis observed in the first 100 days of lactation occurred in quarters, which had an open teat canal 10 days before calving [3]. The findings of the present study are in accordance with the conclusion of Krömker and Friedrich [3] that the avoidance of colonization of the mastitis-causing pathogens on mammary glands could minimize the risk of heifer mastitis.

In the present study, no association between the application of the teat dip and the somatic cell count as an indicator for the inflammatory status of an udder quarter [32] was found despite the observed effects of the application of the teat dip on intramammary infections caused by major pathogens. It is hypothesized that the application of the teat dip may stimulate the immune response, but further research is needed to understand the interactions between the used lactic acid bacteria strains and other components of the teat dip and the host. In several studies, immunomodulatory effects have been reported after the application of lactic acid bacteria into the bovine mammary gland. According to Beecher et al., a localized immune response is stimulated after the infusion of *Lc. lactis* DPC 3147 into the mammary gland [20]. Crispie et al. observed an increase in neutrophils incorporation into the udder within 48 h after the infusion of a live *Lc. lactis* culture [19]. The intramammary infusion of *Lb. perolens* CRL 1724 at drying-off leads to the incorporation of neutrophils to the epithelial zone of the cistern [34].

## 4. Materials and Methods

### 4.1. Herds and Animals

The study was conducted on German Holstein black pied cows in five commercial dairy farms in Northern and Central Germany. The average size of the herds ranged from 154 to 1539 lactating cows, producing average milk yields between 7300 and 11,176 kg per cow. The cattle were housed in free-stall barns with cubicles or in straw-bedded pens. The udder health of the included dairy herds was characterized by a high rate (41% to 61%) of annual heifer mastitis (heifers with more than 100,000 somatic cells/mL at first DHI sampling in all calved heifers per year). No pharmaceutical agent was used for the prevention of heifer mastitis. Only clinically healthy pregnant heifers with four teats appearing functional without injuries or signs of cured injuries were included in the trial.

### 4.2. Experimental Design

The study used a split-udder design. Two weeks before the expected calving date, two researchers, well-trained in treating and sampling procedures, started dipping randomly, based on a computer generated random list, either the left front and the right hind teat or the right front and the left hind teat of dairy heifers in the teat dip suspension containing live lactic acid bacteria once a week until calving. We have chosen a dip interval of one week, as we were able to isolate the relevant microorganisms back from the tip of the teat up to one week after dip in various preliminary tests. A conventional dip cup without back flow was used for all treated teats in one herd. The treated teats were dipped briefly (1 second) in the solution, as with a commercial dipping agent. The teats were not washed, cleaned, or dried before application.

The contralateral teats were not treated with the teat dip during the study period. A treated teat was matched with its contralateral untreated teat to minimize individual animal effects [21].

The foremilk samples were taken aseptically from all quarters of the animals included in the study at day 7 ± 2 (C1) and 14 ± 2 (C2) after calving.

All applicable guidelines for the care and use of animals were followed. The study was approved by the animal welfare committee of the university (Az1623). An application for a license for animal testing was not required by the local government due to the external use of GRAS microorganisms. The study meets the International Guiding Principles for Biomedical Research Involving Animals (1985).

### 4.3. Preparation of Teat Dip

In addition to the standard strains *Lb. rhamnosus* ATCC 7469 and *Lc. lactis* subsp. *lactis* ATCC 11454, two wild strains *Lb. paracasei* 78/37 (DSM 26911) and *Lb. plantarum* 118/37 (DSM 26912) were also used, which were isolated and characterized at the University of Applied Sciences and Arts Hannover by Diepers et al. [17] and Wallis et al. [35]. The noncommercial teat dip suspension was prepared one day before application. The individual strain was cultured in 50 mL MRS broth (Oxoid, Wesel, Germany; the pH was adjusted to 5.5 by adding lactic acid) and incubated at 37 °C for 72 h. Afterwards, 25 mL of the culture was centrifuged at 900 × *g* for 10 min. The bacterial pellet was resuspended in the remaining 25 mL of noncentrifuged culture. The final teat dip suspension contained 19.75% (v/v) of individually enriched strain in MRS broth, 20% of 10% skim milk powder solution, and 1% of a 10% (w/v) filter sterilized chlorophyllin-copper complex solution (Caelo, Hilden, Germany) as a colorant. The concentration of each strain before mixing was determined, and the ratio between the strains was confirmed as equal for each strain. In the final teat dips, the total counts of lactic acid bacteria ranged from 8.40 to 8.47 log_10_-transformed CFU/mL as detected on MRS agar (Applichem, Darmstadt, Germany) after incubation at 37 °C for 48 h.

### 4.4. Sampling Procedure

As mentioned earlier, quarter foremilk samples were collected aseptically at C1 and C2. According to NMC, after cleaning teats with dry paper towels, the first three streams of milk were discarded and the teat ends were disinfected with 70% ethanol [36]. Approximately 10 mL of milk was collected per quarter in duplicate into sterile tubes by two well-trained researchers. The samples were transported at 5 °C to the microbiology lab at the University within eight hours, and were analyzed within two hours of arrival by a trained blinded researcher.

### 4.5. Cyto-Microbiological Analysis and Definitions

For the microbiological diagnosis, 10 µL of a well-mixed quarter foremilk sample was plated onto a quadrant of an aesculin blood agar plate (Oxoid, Wesel, Germany). The plates were incubated aerobically at 37 °C and examined after 24 and 48 h, respectively, following the GVA recommendations [37]. The colonies were identified by Gram staining, cell morphology, aesculin hydrolysis, and hemolysis patterns. Catalase and Gram-positive cocci were identified as *Staph. aureus* characterized by beta-hemolysis and having a positive clumping factor (Diamondial StaphPlus Kit, Diamondial, Sées, France) reaction. Other staphylococci were referred to as NAS. Gram-positive and catalase negative cocci were identified as streptococci. The modified Rambach agar was used to differentiate the aesculin hydrolysing cocci [38]. Aesculin hydrolysing, β-D-galactosidase-positive cocci were identified as *Strep. uberis,* and aesculin hydrolysing, β-D-galactosidase-negative cocci were identified as *Enterococcus* spp. Aesculin-negative streptococci were further characterized by Lancefield serotyping (group B streptococci: *Strep. agalactiae*, group C streptococci: *Strep. dysgalactiae*; Diamondial Strep Kit, Diamondial, Sées, France). Gram-positive, asporogenic, beta-hemolytic, and catalase-negative irregular rods with a V- or Y-shaped configuration were identified as *Trueperella* (*T.*) *pyogenes*. *Bacillus* spp. were Gram-positive and catalase positive rods, which are able to form endospores. Coliform bacteria were Gram-negative and cytochrome oxidase-negative (Bactident oxidase, Merck, Darmstadt, Germany) rod-shaped bacteria that were capable of fermenting glucose (OF basal medium with the addition of D [+]-glucose monohydrate, Merck, Darmstadt, Germany). On ChromoCult® Coliform Agar (Merck, Darmstadt, Germany), *E. coli* formed blue colonies, unlike other coliforms that formed pink-red colonies. Pseudomonads were identified as Gram-negative, catalase positive, cytochrome oxidase-positive rod-shaped bacteria that utilize glucose oxidatively (OF test). Yeasts and *Prototheca* spp. were differentiated microscopically after subculture on YGC agar (Merck, Darmstadt, Germany).

The samples were categorized as positive with a growth of a minimum of five colonies. However, *Staph. aureus*, *Strep. Agalactiae,* and *Strep. dysgalactiae* were identified even if a single colony was detected. If the growth of more than two different types of colonies was identified, the sample was considered to be contaminated. However, the growth of *Staph. aureus* and *Strep. dysgalactiae* was reported from contaminated samples.

Somatic cell counts of the milk samples were determined using a flow cytometer SomaScope Smart (Delta Instruments B.V., Drachten, The Netherlands).

On the basis of the cyto-microbiological investigation of the quarter foremilk samples, a quarter was considered infected if the same pathogen was detected in both C1 and C2 samples [26].

### 4.6. Data Analysis

The data were recorded using Microsoft Excel 2010 software (Microsoft, Redmond, WA, USA). Somatic cell counts were expressed as log_10_-transformed. All statistical analyses were performed with SPSS 26.0 (IBM, Armonk, NY, USA). The associations between the variables IMI (yes/no), IMI caused by a major pathogen (pathogen other than NAS and *Corynebacterium* spp. [32]; yes/no), detection of a major pathogen at C1 or C2, log_10_-transformed somatic cell counts at C1 and C2 and the occurrence of clinical mastitis in the first 100 days after calving (e.g., clots/flakes in milk, swelling/redness of udder; yes/no), and the fixed effects dipping (yes/no) and the number of treatments before calving were investigated with the generalized linear mixed regression models for repeated measurements. The subject was the teat pair consisting of a treated and its contralateral untreated teat. The farm was treated as a random effect, and the statistical significance was accepted at *p* < 0.05.

## 5. Conclusions

In conclusion, the results of the present study indicate that the external application of lactic acid bacteria on teats reduces the risk of IMIs with major pathogens at the beginning of lactation in heifers as well as the risk for clinical mastitis in the first 100 days after calving. However, further research is required to investigate the interactions between the lactic acid bacteria and mastitis-causing pathogens, teat and udder epithelia, and their influence on host immune response.

## Figures and Tables

**Table 1:** Postcalving microbiological results for untreated and treated quarters.

Pathogen Causing Intramammary Infection (IMI)	Untreated Quarters (n = 629) n (%)	Treated Quarters ^a^ (n = 629)				
		1 × Precalving Application n (%)	2 × Precalving Applications n (%)	3 × Precalving Applications n (%)	4 × Precalving Applications n (%)	5 × Precalving Applications n (%)
no growth	585 (93.0%)	245 (93.9%)	237 (92.9%)	99 (100.0%)	10 (100.0%)	4 (100.0%)
NAS ^b^	22 (3.5%)	6 (2.3%)	16 (6.3%)			
*Staph. aureus*	19 (3.0%)	10 (3.8%)	1 (0.4%)			
*Strep. dysgalactiae*	2 (0.3%)					
*Enterococcus* spp.	1 (0.2%)		1 (0.4%)			
Total	629	261	255	99	10	4

a: one application of the teat dip per week; starting two weeks before the expected calving date. b: nonaureus staphylococci/coagulase-negative staphylococci.
